# Purification and Characterization of a Mitogenic Lectin from *Cephalosporium*, a Pathogenic Fungus Causing Mycotic Keratitis

**DOI:** 10.1155/2010/854656

**Published:** 2010-04-06

**Authors:** Nagaraja N. Nagre, Vishwanath B. Chachadi, Sachin M. Eligar, C. Shubhada, Radha Pujari, Padma Shastry, Bale M. Swamy, Shashikala R. Inamdar

**Affiliations:** ^1^Department of Biochemistry, Karnatak University, Dharwad 580 003, Karnataka, India; ^2^National Center for Cell Sciences, Ganeshkhind, Pune 411 007, India

## Abstract

Ophthalmic mycoses caused by infectious fungi are being recognized as a serious concern since they lead to total blindness. *Cephalosporium* is one amongst several opportunistic fungal species implicated in ophthalmic infections leading to mycotic keratitis. A mitogenic lectin has been purified from the mycelia of fungus *Cephalosporium*, isolated from the corneal smears of a keratitis patient. *Cephalosporium* lectin (CSL) is a tetramer with subunit mass of 14 kDa, agglutinates human A, B, and O erythrocytes, and exhibits high affinity for mucin compared to fetuin and asialofetuin but does not bind to simple sugars indicating its complex sugar specificity. CSL showed strong binding to normal human peripheral blood mononuclear cells (PBMCs) to elicit mitogenic activity. The sugar specificity of the lectin and its interaction with PBMCs to exhibit mitogenic effect indicate its possible role in adhesion and infection process of *Cephalosporium*.

## 1. Introduction

Ophthalmic infections caused by fungi rather than bacteria are of serious concern in many developing countries as they lead to blindness. Ocular fungal infections referred to as ophthalmic mycoses are leading to keratitis of the cornea [1]. Several fungal species responsible for mycotic keratitis and other forms of eye infections have been reviewed recently [2, 3]. *Fusarium *and *Cephalosporium* (*Acremonium*) are wide spread fungal species that belong to hyaline filamentous group which are implicated for ophthalmic mycoses [3]. The key factors involved in the pathogenesis of the mycotic keratitis include adherence, invasiveness, morphogenesis, and toxigenicity [4]. Fungal lectins are gaining importance as they are implicated in the process of specific recognition between fungal parasites and their host cells, involving protein-carbohydrate interactions leading to infection [5]. Lectins constitute a heterogeneous group of proteins of nonimmune origin with noncatalytic binding sites capable of recognizing and binding reversibly to specific carbohydrate moieties [6]. Although several reports are available on the lectins from higher fungi [7], very few lectins from animal and plant pathogenic fungi are reported [5, 8–13]. Further, fungal lectins are drawing greater attention as many of them exhibit interesting physiological effects such as lymphomitogenic activity, immunomodulatory effect, suppression of cell proliferation, and antitumor activity [14].

The present paper reports the purification, characterization, and mitogenic activity towards human PBMCs of a lectin from *Cephalosporium,* a pathogenic fungus, isolated from the infected eye of a keratitis patient. 

## 2. Materials and Methods

### 2.1. Fungal Culture

The fungus was isolated from the corneal smears of a female patient aged 48 years with fungal keratitis. The corneal scrapings were inoculated on Sabourd's dextrose agar (SDA) slants and the stock culture was maintained on SDA slants. *Cephalosporium *cultures were grown in 500-ml Erlenmeyer flasks containing 100 ml Byrde's liquid synthetic media (0.696 g KH_2_PO_4_, 0.149 g KCl, 0.008 g ZnSO_4_·2H_2_O, 0.2 g MgSO_4_, 0.2 g FeSO_4_, 0.006 g MnSO_4_, and 1 g NH_4_NO_3_ per liter of distilled water) with 2% dextrose and incubated at room temperature under stationary conditions [15]. After 10 days, the mycelial mat was harvested and washed with distilled water on cheese cloth. The mycelial mass was subsequently freeze dried and powdered in a glass mortar, and the fine powder was stored at −20°C and used for the purification of lectin. 

### 2.2. Materials

Mucin (porcine stomach, type III), fetuin (fetal calf serum), PHA-L, Histopaque 1077, and fine sugars used in hapten inhibition studies were purchased from Sigma Chemical Co., St. Louis, USA. Sepharose 4B was obtained from Pharmacia Fine Chemicals, Uppsala, Sweden. Asialofetuin was prepared as described by Spiro and Bhoyroo [16]. Asialofetuin-Sepharose 4B, affinity matrix, was prepared by coupling asialofetuin to cyanogen bromide-activated Sepharose 4B according to the method of March [17]. Tritiated thymidine was procured from BRIT (Board of Radiation and Isotope Technology), India. The tissue culture flasks and 96 well plates were procured from NUNC (Denmark). Human blood samples were obtained from Health Center, Karnatak University, Dharwad, India, with approval from Institutional Review Board. All other chemicals used were of analytical reagent grade. 

### 2.3. Isolation and Purification of CSL

Dried *Cephalosporium* mycelial powder was homogenized in 50 ml (1 : 50 w/v) of 50 mM sodium phosphate buffer, pH 7.2, containing 154 mM NaCl (PBS) for 5 minutes and stirred overnight at 4°C. The extract was centrifuged (9500 xg) for 30 minutes at 4°C. The resulting supernatant was membrane filtered (0.45 *μ*m) and used for the purification of the lectin. The crude extract was subjected to affinity chromatography on asialofetuin-Sepharose 4B column (10 × 1.3 cm) that had been equilibrated with PBS and 3 ml fractions were collected at a flow rate of 15 ml/hr. The washing of the column with PBS was continued, until the absorbance of the eluting fractions reads zero at 280 nm (double beam spectrophotometer, Hitachi 2800). The bound lectin was eluted with glycine-HCl buffer (100 mM, pH 2.0) containing 500 mM NaCl. Fractions containing lectin activity were pooled and dialyzed extensively against PBS and stored at −20°C for further studies.

### 2.4. Hemagglutination Assay and Hapten Inhibition Studies

Hemagglutination activity of CSL during various stages of purification was determined by the serial twofold dilution method using trypsinized human erythrocytes in 96 well, “U” bottom micro titer plates [18]. The highest dilution of the extract causing visible hemagglutination was regarded as the titre and the minimum concentration of the protein was required for agglutination (MCA) as one unit of hemagglutinating activity. The specific hemagglutination activity was expressed as unit mg^−1^ protein. The sugar specificity of the purified lectin was determined by a hapten inhibition assay. Inhibition assays were carried out by incubating the lectin sample (with hemagglutinating titre of 4) with serially diluted sugar/glycoprotein prior to the addition of erythrocytes in a total volume of 50 *μ*l, and the hemagglutination was visually observed. The minimum inhibitory concentration of the sugar/glycoprotein was taken as the inhibitory titre of the hapten. 

Protein concentrations were determined routinely by Lowry's method [19] and total sugar content of the lectin was estimated by phenol-sulfuric acid method [20].

### 2.5. Molecular Mass Estimation

The molecular mass of the purified lectin was determined by SDS-PAGE in 15% gel, according to the method of Laemmli [21] and by gel filtration chromatography on a superdex G-75 column (1.5 × 80 cm, in PBS) precalibrated with standard proteins of known molecular weight.

### 2.6. Binding of CSL to Human PBMCs

Human peripheral blood mononuclear cells (PBMCs) were isolated from the blood of healthy donors by density gradient using Histopaque-1077 (Sigma) and resuspended in complete medium (RPMI 1640 + 10% FCS). Fluorescein isothiocyanate-conjugated CSL (FITC-CSL) required for flow cytometry was prepared using the protocol of Goldman [22]. The binding of CSL to PBMCs was determined by flow cytometry. Cells (1 × 10^5^) were incubated with FITC-CSL (2 *μ*g/100 *μ*l) for 1 hour on ice and were washed thoroughly with 50 mM PBS and then fixed with 2% freshly prepared paraformaldehyde. Data were acquired for 10,000 events using an FACS Vantage (Becton Dickson) equipped with a 488 nm argon laser and analyzed with Cellquest-pro software for determining % positivity and mean fluorescence intensity (MFI). Unstained cells that had been processed similarly were used as negative control.

Receptor-mediated CSL binding to PBMCs was determined by preincubating FITC-CSL with mucin, fetuin and asialofetuin (100 *μ*g/ml) for 1 hour at room temperature. This lectin-sugar complex was added to the PBMCs preparation and analyzed by flow cytometry.

### 2.7. Mitogenic Activity by Tritiated Thymidine Incorporation Assay

Freshly isolated PBMCs were suspended in RPMI-1640 containing 10% FCS and 1 × 10^5^ cells/100 *μ*l/well were plated in 96 well tissue culture plate (NUNC, Denmark) and incubated with CSL concentration ranging from 0.625 *μ*g/ml to 10 *μ*g/ml for 72 hours at 37°C in 5% CO_2_. PBMCs stimulated with PHA-L (0.156 *μ*g/ml to 2.5 *μ*g/ml, Sigma Chemicals) were used as positive control. Cells were pulsed with tritiated thymidine (1 *μ*Ci /well, BRIT, India) 18 hour prior to harvesting and incorporation was measured as counts per minute (CPM).

### 2.8. Statistical Analysis

Statistical analysis was performed using student's *t*-test and Mann-Whitney rank sum test. A *P*-value <  .05 was considered to be statistically significant.

## 3. Results

### 3.1. Isolation and Purification of the Lectin from the Mycelium of Cephalosporium

**Figure 1:**
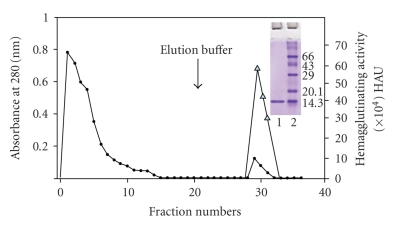
Affinity purification of *Cephalosporium* lectin on asialofetuin-Sepharose 4B column. Crude extract was passed through affinity column, equilibrated in PBS, and the bound lectin was eluted with elution buffer. Fractions of 3.0 ml were collected at a flow rate of 15 ml/hr. 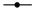 Absorbance at 280 nm, 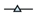 Hemagglutinating activity. Inset- SDS-PAGE of affinity purified CSL in 15% gel. The purified lectin (30 *μ*g) is indicated in lane 1 and lane 2 contains standard molecular weight markers. The gel was stained with Coomassie brilliant blue.

**Figure 2:**
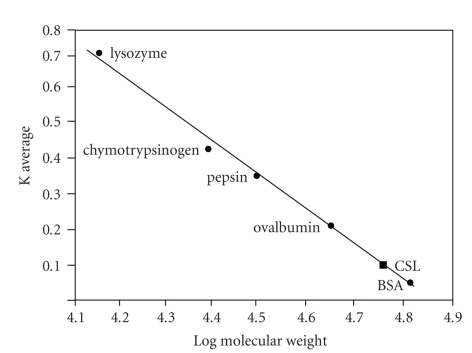
Calibration curve for the estimation of molecular weight of CSL by gel filtration chromatography. *X*-axis represents the log molecular weight and *Y*-axis represents K average, marker proteins; BSA (66 kDa), ovalbumin (45 kDa), pepsin (34.7 kDa), chymotrypsinogen (25.6 kDa) and lysozyme (14.3 kDa).

The lectin was purified to homogeneity in a single step by affinity chromatography on asialofetuin-Sepharose 4B column (Figure 1). The fold purification and the total % recovery of the purified lectin from 1 g of the dry mycelial powder are summarized in Table 1; the minimum concentration of the protein required for agglutination (MCA) was found to be 1.145 *μ*g for the crude extract and 0.021 *μ*g for the purified lectin. The eluted lectin was found to be homogenous as revealed by single band on SDS-PAGE in 15% gel (Figure 1, Inset). Subunit molecular mass of 14 kDa was estimated for the lectin by SDS-PAGE, whereas molecular mass of 57 kDa was estimated by gel filtration chromatography (Figure 2), suggesting tetrameric nature of the lectin. Purified CSL agglutinated the human erythrocytes of all blood groups, indicating that it has a blood group nonspecific nature. Hapten inhibition studies showed that the hemagglutinating activity of CSL was inhibited by mucin, fetuin, and asialofetuin, with mucin being the most potent inhibitor with minimum inhibitory concentration (MIC) of 0.785 *μ*g/50 *μ*l (Table 2). 

### 3.2. Binding of CSL to Human PBMCs

**Figure 3:**
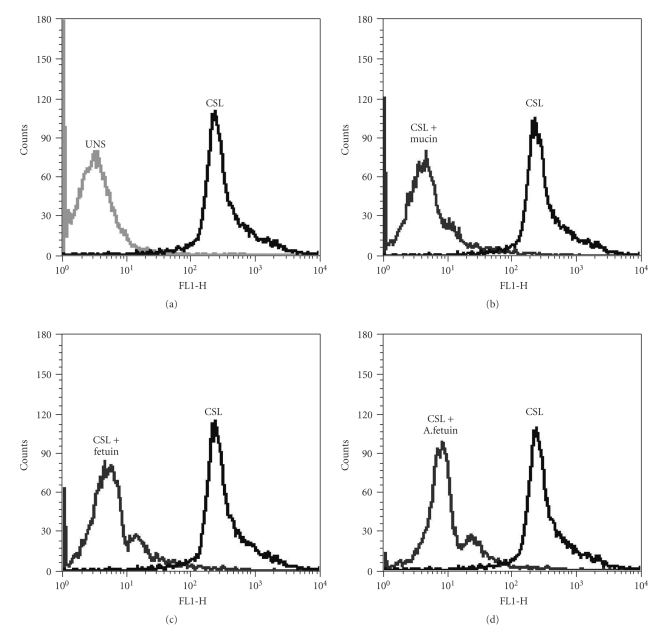
Binding of CSL to human PBMCs and inhibition of binding with competing glycoconjugates. PBMCs were stained with FITC-labeled CSL and subjected to flow cytometric analysis. *X*-axis, FL1-H on a log scale represents the fluorescence intensity of cells stained with FITC labeled CSL. *Y*-axis represents cell number. (a) The histoplot shows profiles of the unstained cells (UNS) and cells stained with FITC-labeled CSL (CSL). Profiles of cells stained with FITC-labeled CSL preincubated with different haptens are indicated in (b, c and d).

To study the interaction of CSL with PBMCs, cells were stained with FITC-CSL and its binding was analysed by flow cytometry. As depicted in Figure 3(a), 99.62% of the cells were found to be positive for CSL binding, with mean fluorescence intensity (MFI) of 411.27, in comparison with control unstained cells which were set to 1% positivity and the MFI of this 1% cells was 76.89. The receptor-mediated lectin binding to PBMCs was confirmed by studying the binding of CSL after pre-incubation of the lectin with different competing glycoconjugates or haptens. Flow cytometry histograms of CSL binding to PBMCs before and after blocking with mucin, fetuin, and asialofetuin are presented in Figures 3(a), 3(b), 3(c), and 3(d), respectively. Mucin at 100 *μ*g/ml concentration was the most effective inhibitor of CSL followed by fetuin and asialofetuin at 100 *μ*g/ml.

### 3.3. Mitogenic Activity of CSL

**Figure 4:**
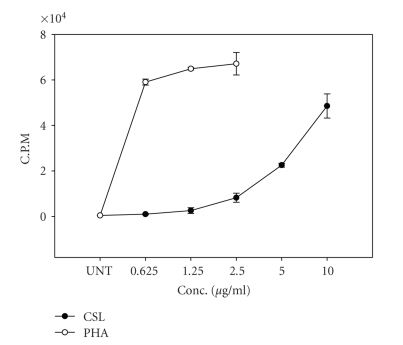
Mitogenic activity of CSL. PBMCs were isolated from blood of healthy donors and exposed to serial concentrations of CSL (0.625–10 *μ*g/ml) and PHA-L (0.16–2.5 *μ*g/ml) for 72 hours and proliferation was measured by tritiated thymidine incorporation assay as counts per minute (CPM). The data are presented as mean ± SE of four independent experiments done in triplicates.

Mitogenic effect of CSL on human PBMCs was determined by tritiated thymidine incorporation assay. CSL stimulated the uptake of thymidine by human PBMCs in a dose-dependent manner, with maximum incorporation occurring at 10 *μ*g/ml of the different doses tested (0.625–10 *μ*g/ml), whereas PHA-L, a positive control exhibited maximum proliferation at 1.25 *μ*g/ml (Figure 4).

## 4. Discussion

In the present study, the lectin from human pathogenic fungus *Cephalosporium* isolated from mycotic keratitis patient was purified to homogeneity in a single step by affinity chromatography on asialo fetuin-Sepharose 4B. CSL, a tetramer, without blood group specificity, has complex sugar specificity and mitogenic activity on human PBMCs. In the past decade, sizeable numbers of fungal lectins are reported and majority of them are from higher fungi, mostly from fruiting bodies of mushrooms [7, 23]. However, very few lectins are known from the mycelia forming lower fungi, particularly the pathogenic fungi, although fungal lectins are implicated in host parasite interactions [5]. There is a growing realization that the fungal lectins like bacterial lectins also play an important role in adhesion and infection process. Several hyaline filamentous fungi are considered as opportunistic human pathogens [3]. Most of these pathogens express surface factors, lectins that mediate binding to extra cellular matrix glycoproteins of host cells. Such a binding would facilitate the fungal adherence and can induce immunomodulatory effect on the host cells. In this regard, the CSL exerts mitogenic activity on human PBMCs. So far, very few lectins from human pathogenic fungi are reported including *Candida albicans*, *Candida glabrata*, *Histoplasma capsulatum, Aspergillus fumigatus*, and *Trycophyton rubrum *[5, 10, 24, 25]. The lectin from *C. albicans* which recognizes Fuc*α*1-2Gal*β*, occurring on all blood group substances of ABO types, is implicated in the adherence to human buccal epithelial cells [9]. A 32 kDa lectin purified from *A. fumigatus*, with sialic acid specificity is suggested to play an important role in the attachment of conidia to extra cellular matrix components of host cells [10]. *A. fumigatus *is a known opportunistic pathogen responsible for variety of ophthalmic infections such as dacryocystitis, scleritis and endophthalmitis [3]. An extracellular agglutinin from *Trycophyton rubrum, *a well-known dermatophyte, has specificity towards sialo oligosaccharides is implicated for the adhesion to host cells [25].

 There is no direct evidence available confirming the role of lectins from these pathogens in mediating host parasite interactions and also in their immunomodulatory effects on the host cells. On the contrary, several lectins from higher fungi are known to exert interesting immunomodulatory effects. Lectins from* V. volvacea* and *Boletus satanas* Lenz exhibit mitogenic effects on human PBMCs [26, 27], whereas *Agrocybe cylindracea* and *Schizophyllum commune* exerts mitogenic effect on mouse splenocytes [28, 29]. Some of the mitogenic lectins like those from *V. volvacea, F. velutipes,* and *Ganoderma lucidum *were shown to exhibit immunomodulatory effects [30–32], and such mitogenic lectins have potential applications as important diagnostic and experimental tool to study the various aspects of cell growth and differentiation. The receptor mediated binding of CSL to human PBMCs was clearly demonstrated, as it can be effectively blocked by competing glycoconjugates such as mucin, fetuin and asialofetuin. These results substantiate our observations by hapten inhibition studies that CSL has preferential affinity towards mucin compared to fetuin or asialofetuin. The mitogenic potential of CSL was demonstrated by the observed dose-dependent increase in proliferation of PBMCs. 

In summary, the lectin with complex sugar specificity purified from the pathogenic fungus *Cephalosporium,* which is responsible for ophthalmic mycoses, exhibits mitogenic and hemagglutinating activity. It is one of the few lectins characterized from pathogenic fungi reported and the information provides valuable account for understanding the lectin mediated host-parasite interaction in fungal infections.

## Figures and Tables

**Table 1:** Purification of CSL from mycelial extract.

Sample	Volume (ml)	Protein (mg)	Sugar (mg)	MCA^a^ (*μ*g)	Specific activity^c^ (units)	Total activity^d^ (units)	Fold purification	Recovery of activity (%)
Original crude extract	41.0	30.053	22.96	1.145	0.087 × 10^4^	2.61 × 10^4^	—	100
Affinity purified	7.5	0.410	ND^b^	0.021	4.76 × 10^4^	1.95 × 10^4^	54.71	74.71

^a^-Minimum concentration of protein required to agglutinate erythrocytes used.

^b^-Not detected by phenol-sulfuric acid method.

^c^-Specific activity: hemagglutinating activity mg^−1^ protein.

^d^-Total activity: hemagglutinating activity of lectin in total protein.

**Table 2:** Hapten inhibition studies with purified CSL.

Glycoprotein	Minimum concentration required for inhibition, MIC (*μ*g/50 *μ*l )
Mucin (from porcine stomach)	0.785
Fetuin	6.25
Asialofetuin	12.5

*D-(+)-Galactose, D-(+)-Glucose, D-(+)-Mannose, D-(+)-Arabinose, D-(+)-Fucose, L-(-)-Fucose, *β*-D(+)-Glucose, 2-Deoxy-D-glucose, *α*-L-Rhamnose, 1-Amino-1-deoxy-*β*-D-glucose, N-Acetyl-D-galactosamine, N-Acetyl-D-glucosamine, N-Acetyl-*β*-D-mannosamine, Methyl-*α*-D-mannopyranoside, Methyl-*α*-D-galactopyranoside, Methyl-*β*-D-galactopyranoside, Methyl-*β*-D-glucopyranoside, 4-Aminophenyl *β*-D-galactopyranoside, 4-Aminophenyl *β*-D-glucopyranoside, N-Acetyl neuraminic acid, *β*-D-Galactose(1-4) *β*-D-glucose (*β*-Lactulose), *β*-D-Glucose(1-4)-D-glucose (cellobiose), 6-*α*-D-Galactopyranosyl-D-glucopyranose (melibiose), and O-*α*-D-Galactopyranosyl-(1-6)-*α*-D-glucopyranosyl-*β*-D-fructofuranoside (raffinose) did not inhibit the lectin activity up to concentrations of 200 mM. Also, ovalbumin (2 mg/ml), and the plant polysaccharides guar gum (1 mg/ml), gum acacia (10 mg/ml), were not inhibitory.
